# A multiscale X-ray phase-contrast tomography dataset of a whole human left lung

**DOI:** 10.1038/s41597-022-01353-y

**Published:** 2022-06-02

**Authors:** R. Patrick Xian, Claire L. Walsh, Stijn E. Verleden, Willi L. Wagner, Alexandre Bellier, Sebastian Marussi, Maximilian Ackermann, Danny D. Jonigk, Joseph Jacob, Peter D. Lee, Paul Tafforeau

**Affiliations:** 1 Department of Mechanical Engineering, University College London, London, UK; 2 Antwerp Surgical Training, Anatomy and Research Centre (ASTARC), University of Antwerp, Wilrijk, Belgium; 3 Department of Diagnostic and Interventional Radiology, University Hospital Heidelberg, Heidelberg, Germany; 4 Translational Lung Research Centre Heidelberg (TLRC), German Lung Research Centre (DZL), Heidelberg, Germany; 5 Laboratoire d’Anatomie des Alpes Françaises (LADAF), Université Grenoble Alpes, Grenoble, France; 6 Institute of Pathology and Molecular Pathology, Helios University Clinic Wuppertal, University of Witten/Herdecke, Wuppertal, Germany; 7 Institute of Functional and Clinical Anatomy, University Medical Center of the Johannes Gutenberg-University Mainz, Mainz, Germany; 8 Institute of Pathology, Hannover Medical School, Hannover, Germany; 9 Biomedical Research in End-stage and Obstructive Lung Disease Hannover (BREATH), German Lung Research Centre (DZL), Hannover, Germany; 10 Centre for Medical Image Computing, University College London, London, UK; 11 Department of Radiology, University College London Hospitals NHS Foundation Trust, London, UK; 12 European Synchrotron Radiation Facility, Grenoble, France

**Keywords:** Anatomy, Respiration, X-ray tomography

## Abstract

Technological advancements in X-ray imaging using bright and coherent synchrotron sources now allows the decoupling of sample size and resolution while maintaining high sensitivity to the microstructures of soft, partially dehydrated tissues. The continuous developments in multiscale X-ray imaging resulted in hierarchical phase-contrast tomography, a comprehensive approach to address the challenge of organ-scale (up to tens of centimeters) soft tissue imaging with resolution and sensitivity down to the cellular level. Using this technique, we imaged *ex vivo* an entire human left lung at an isotropic voxel size of 25.08 *μ*m along with local zooms down to 6.05–6.5 *μ*m and 2.45–2.5 *μ*m in voxel size. The high tissue contrast offered by the fourth-generation synchrotron source at the European Synchrotron Radiation Facility reveals the complex multiscale anatomical constitution of the human lung from the macroscopic (centimeter) down to the microscopic (micrometer) scale. The dataset provides comprehensive organ-scale 3D information of the secondary pulmonary lobules and delineates the microstructure of lung nodules with unprecedented detail.

## Abstract (summary)

**Table:** 

Measurement(s)	Human left lung
Technology Type(s)	X-Ray Phase-Contrast Imaging

## Background & Summary

The human lung is among the largest solid organs in the human body. Traditionally, studies of lung microanatomy at the organ scale require lengthy operations in targeted sampling, tissue preparation, histological staining, and sectioning^1,2^. Nowadays, *ex vivo* clinical evaluations of whole lung microstructures are carried out without sectioning using absorption-contrast micro-CT at around 100 *μ*m voxel size. A limited area of the lung may then be selected for imaging at higher resolution using histology^3–5^. X-ray phase-contrast imaging^6,7^ provides higher sensitivity and contrast than laboratory micro-CT^8^. Compared with optical virtual histology^9^, X-ray phase contrast from free-space propagation requires no imaging optics and, at the same time, removes the need for laborious tissue clearing and fluorescent labeling that are essential for optical imaging^10^. The compatibility of X-ray phase-contrast imaging with existing X-ray sources will facilitate its gradual adoption and transition from preclinical research to clinical diagnostics^6,11,12^. At synchrotron facilities, systematic upgrades^13,14^ in the X-ray source and imaging techniques over the past decades provide the means to tackle biological questions across meaningful scales and resolution^11,15–20^. Although synchrotron-based X-ray imaging can access finer anatomical detail than laboratory micro-CT^19,21–23^, many bioimaging scenarios require further upscaling of the imaging throughput and accommodation of large sample size while maintaining microscopic resolution^24,25^.

**Fig. 1:**
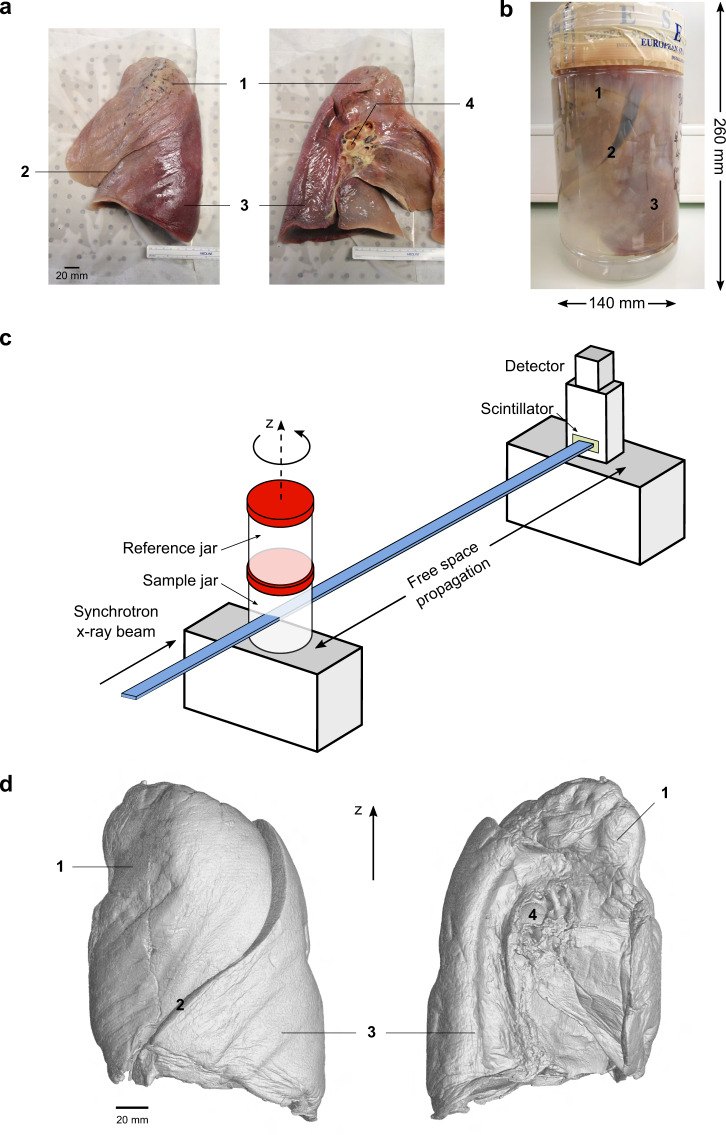
(**a**) A human left lung in lateral (left) and medial (right) view. (**b**) The instillation-fixed whole lung is mounted in a sealed, size-compatible, plastic cylindrical PET jar (140 mm in diameter, 260 mm in height) filled with 70% ethanol solution and agar blocks. (**c**) Sketch of the HiP-CT imaging setup using propagation phase contrast from bright and coherent X-rays at ESRF BM05 beamline. The reference jar contains the same embedding medium as the sample jar. The incident X-ray energy is adjusted to 70–85 keV through filters depending on the resolution requirement. (**d**) Volume rendering of the whole left lung imaged at 25.08 *μ*m voxel size using HiP-CT in lateral (left) and medial (right) views. Major anatomical features labeled in **a**,**b**,**d** are the (1) left upper lobe, (2) interlobar fissure, (3) left lower lobe, (4) left mainstem bronchus.

Thanks to the high X-ray photon flux and spatial coherence achieved at modern fourth-generation synchrotron sources and careful design of the sample preparation and imaging protocol, it is now possible to image complete, large, partially dehydrated human organs in their entirety at micrometer resolution using hierarchical phase-contrast tomography (HiP-CT)^26^. The technique integrates a multiscale imaging workflow^4,27–30^ into a single setup, utilizing propagation phase contrast obtained from high-energy, polychromatic X-rays and tunable detection settings. Therefore, scanning an entire human organ (with a size of 5–30 cm in each dimension) at multiple resolutions can be executed without dissecting the sample or requiring to transport to different instrument locations or facilities^27,28,30^. HiP-CT features a customized flat-field correction, an attenuation scanning protocol, along with an efficient tomographic sampling and stitching pipeline to cover large, soft-tissue organs entirely, without staining^27,31^ or clearing^10^. The integrated single-modality, multiscale imaging approach of HiP-CT^26^ ensures a simplified image registration procedure thanks to the consistent tissue contrast across lengthscales. Its imaging protocol takes inspiration from existing multiscale approaches^4,18,19,29^, starting with a two-step tomographic sampling of the whole organ (full-field tomography), followed by progressive zoom-ins to selected features of the microanatomy through local tomographies at various finer resolutions compatible with the relevant anatomical context. HiP-CT requires the sample such as a soft-tissue organ to be embedded in 70% ethanol solution in water and immobilized with agar blocks throughout imaging (see Fig. 1a,b). The flat-field correction takes reference from a separate container (reference jar) of the same size as the sample jar to enhance the soft tissue contrast (see Fig. 1c). We provide here the dataset of an intact human left lung imaged by HiP-CT at 25.08 *μ*m voxel size (full organ, see Fig. 1d) and at 6.05–6.5 *μ*m and 2.45–2.5 *μ*m voxel sizes for various local volumes of interest (VOIs) accomplished by optimization of the incident X-ray spectrum, propagation distance, scintillator thickness, and the coupling optics before the detector (see Methods). The X-ray imaging experiments were carried out at the European Synchrotron Radiation Facility (ESRF) BM05 beamline using the recently upgraded fourth-generation extremely brilliant X-ray source (ESRF-EBS)^32,33^.

## Methods

### Lung preparation and mounting

The entire left lung (see Fig. 1a) was harvested from a body donor, a 94-year-old woman who succumbed to natural causes, with the medical record provided in Table 1. Body donation was based on free consent by the donor antemortem. The relevant postmortem medical procedures were carried out at Laboratoire d’Anatomie des Alpes Françaises (LADAF) according to the Quality Appraisal for Cadaveric Studies scale recommendations^34^. All dissections respected the memory of the deceased. The protocols for transport and imaging were approved by the French legislation for body donation. The body of the deceased donor was embalmed and the lung preparations were carried out at ~36 hours postmortem. The lung was instilled through the trachea with a 4% formalin solution using 30 cm of water column positive pressure. The trachea was then ligatured to maintain the inflated configuration in order to fix the lungs in a non-collapsed state. The body was then kept at 4 °C for 3 days before the dissection. Once removed, the lungs were immersed in 4% formalin solution for 3 more days. Afterwards, it was successively immersed in ethanol solutions (odorless bioethanol, Cheminol, France) with increasing concentration up to 70% (volume fraction). The lung was kept inflated during ethanol dehydration by repeatedly pushing the solution through its main bronchus with a syringe. The significantly lower density of ethanol (789 kg/m^3^) compared with water (1000 kg/m^3^) provides a high base contrast for soft tissues^35,36^.

**Table 1:** Medical record of the body donor.

Donor institution	Laboratoire d’Anatomie des Alpes Françaises
Location	Grenoble, France
Donor ID	LADAF-2020-27
Age	94
Sex	Female
Weight	45 kg
Height	140 cm
Ethnicity	Caucasian
Time of death	2020
Cause of death	Natural cause
Smoking history	No
Donor medical information	1. Right sylvian and right cerebellar stroke
	2. Cognitive disorders of vascular origin
	3. Depressive syndrome
	4. Atrial fibrillation and hypertensive heart disease
	5. Micro-crystalline arthritis (gout)
	6. Right lung pneumopathy (3 years before death)
	7. Cataract of the left eye
	8. Squamous cell carcinoma of the skin (left temporal region)

We used a PET (polyethylene terephthalate) jar of comparable size to the lung for X-ray imaging due to its commercial availability (3600 mL Sweep Jars with Cap, Medline Scientific, UK), high radiation tolerance^37^ and optical transparency in assisting sample alignment and assessment of sample condition during imaging. To secure the lung tightly in place and prevent it from touching the container edges on all sides, we prepared agar (Agar-Agar powder, from wild red seaweed, Nat-Ali, France) blocks in ~1 cm^3^-sized cubes and stacked them at the bottom of the jar and around the organ to firmly embed the lung. The procedure for the agar preparation has been thoroughly described preivously^26^. The gaps between the small agar blocks provide the escape routes for residual gas removal. The sample mounting procedure involves alternated filling of the agar-ethanol mixture and gentle vacuum degassing to minimize the existing microbubbles from dissolved air in the solution environment and within the organ, thereby eliminating their interference with imaging. The degassing procedure used a membrane pump to directly pump^38^ above the PET sample jar with the lid open in a sealed vacuum glass dryer. Prior to imaging, the PET jar containing the lung, ethanol solution, and agar embedding was placed in a custom-made sample holder to connect to the rotation stage at the synchrotron beamline^26^.

### Synchrotron X-ray imaging and reconstruction

**Fig. 2:**
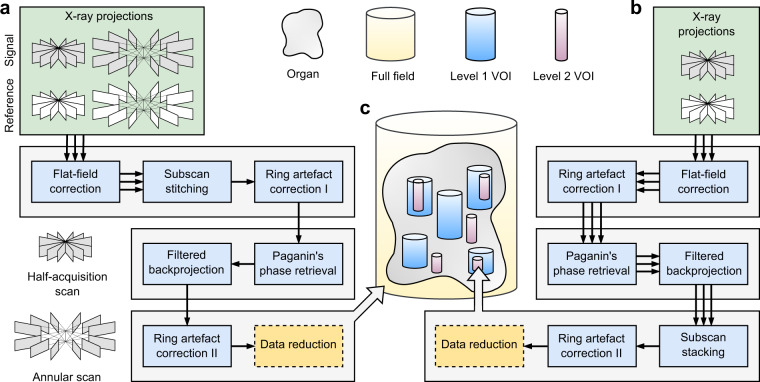
Synchrotron-based hierarchical phase-contrast tomography (HiP-CT) at multiple lengthscales and their associated data acquisition and image reconstruction pipelines. The full-field tomography data at 25.08 *μ*m voxel size are processed with pipeline (**a**). The local tomography data for volumes of interest (VOIs) at 6.05–6.5 *μ*m (level 1) and 2.45–2.5 *μ*m (level 2) voxel size are processed with pipeline (**b**). (**c**) A schematic illustrating the relationships of the various cylindrical volumes imaged with HiP-CT. The triple arrows in the pipeline before merging the subscans indicate that the same procedure is carried out on each subscan.

The implementation and capabilities of HiP-CT have been described in detail in a separate publication^26^. Here, we describe the settings used for lung imaging. All X-ray imaging experiments were carried out at the ESRF bending magnet beamline BM05^39^. The polychromatic synchrotron beam produced at the beamline was passed through a set of filters and then directly used for imaging without additional X-ray optics. The voxel size is effectively controlled by the adjustable visible-light imaging optics situated after the LuAG:Ce (cerium-doped lutetium aluminium garnet) X-ray scintillator (custom-made by Crytur, Czechia) and before the sCMOS (scientific complementary metal-oxide-semiconductor) light sensor (PCO edge 4.2 CLHS, PCO Imaging, Germany). Specifically, the imaging optics include the dzoom (“demagnifying zoom”) and zoom lenses, which cover the ranges of 6.5–25.5 *μ*m and 1.3–6.3 *μ*m, respectively. Because the synchrotron beam size (with usable area 50 mm × 4 mm at BM05) is considerably smaller than the size of the human left lung (container size 260 mm height, up to 140 mm width at the widest), imaging the entire left lung at 25.08 *μ*m voxel size required stitching together multiple subscans. We used the half-acquisition (or half-object acquisition)^40^ method developed at ESRF for imaging the VOIs at 6.5 *μ*m and 2.5 *μ*m in voxel size. For the entire lung, we developed a quarter-acquisition method^26^ that includes the half-acquisition in combination with an annular scan to cover its complete horizontal extent (see Fig. 2).

Data processing of the measured X-ray projections consists of three stages, pre-reconstruction, reconstruction and post-reconstruction, which are illustrated in separate rows in Fig. 2. Ring artifacts from the detectors are corrected in two steps: (1) Before reconstruction, the mean of the projections is subtracted from the projections to remove the rings with constant intensity rings; (2) After reconstruction, the residual inhomogeneous intensity rings were removed using the polar transform combined with linear motion blurring filter^41^. Tomographic reconstruction employs the phase and amplitude estimates obtained from Paganin’s method^42^, followed by a 2D unsharp mask of the retrieved phase maps as input for the filtered backprojection algorithm. These reconstruction steps are implemented in PyHST2^43^. Eventually, the processed volumes are converted to a 16-bit format and binned further to produce the datasets described in Tables 2–3. The reconstruction and postprocessing steps are illustrated for the three types of imaged sample volumes, respectively, in Fig. 2. We summarize below the imaging and reconstruction protocols for the human lung at each imaged resolution including the key parameters.

- Full-field tomography (the whole organ at 25.08 *μ*m voxel size, see Fig. 2a,c): The detected X-ray energy averaged at ~93 keV after filters and sample, the propagation distance was 3475 mm, the scintillator thickness was 2 mm. In total, two sets of 9990 projections were measured by the quarter-acquisition method^26^ with an offset of 800 pixels for the half-acquisition. A step size of 2.2 mm in the vertical (z) direction was used to cover the height of the sample jar with a total of 98 quarter-acquisition subscans. Radiographic stitching was first carried out to recover a half-acquisition scan^40^ before the reconstruction.
- Local tomography of level 1 VOI (6.5 *μ*m and 6.05 *μ*m voxel size, see Fig. 2b,c): The detected X-ray energy averaged at ~88 keV (~89 keV) after filters and sample, the propagation distance was 3500 mm (3475 mm), the scintillator thickness was 1 mm (2 mm) for the VOIs with 6.5 *μ*m (6.05 *μ*m) voxel size. In total, 6000 projections were measured by the half-acquisition method with an offset of 900 pixels. A step size of 2.2 mm in the vertical direction was used to cover the height of the VOIs.
- Local tomography of level 2 VOI (2.5 *μ*m and 2.45 *μ*m voxel size, see Fig. 2b,c): The detected X-ray energy averaged at ~77 keV (~79 keV) after filters and sample, the propagation distance was 1440 mm (1500 mm), the scintillator thickness was 0.25 mm (0.2 mm) for the VOIs with 2.5 *μ*m (2.45 *μ*m) voxel size. In total, 6000 projections were measured by the half-acquisition method with an offset of 900 pixels. A step size of 1.5 mm in the vertical direction was used to cover the height of the VOIs.

**Table 2:** Volumes of interest and their anatomical references to the whole human left lung sample.

VOI reference	Height (mm)	Diameter (mm)	Volume (mL)	Displacement (mm)	Z-axis rotation	Anatomical reference
6.5 µm, VOI1	48.79	25.06	24.1	(23.3, −9.4, −81.2)	24.6°	upper lobe, apical region
6.5 µm, VOI2b	26.74	25.05	13.2	(5.8, −15.0, −26.5)	24.6°	upper lobe, medial region
6.5 µm, VOI3b	9.11	25.04	4.49	(5.5, 0.8, −15.2)	24.6°	interlobar fissure
6.5 µm, VOI4b	106.07	25.06	53.2	(−20.6, 8.1, 37.1)	24.6°	lower lobe, basal to medial region
6.5 µm, VOI5	59.81	25.04	29.5	(−3.3, −15.1, −71.0)	24.6°	upper lobe, apical region
6.05 µm, VOI1	7.61	23.12	3.19	(−12.5, −23.5, −67.9)	2°	upper lobe, apical region
6.05 µm, VOI2	42.17	23.1	17.7	(−7.0, 11.4, 65.5)	0°	lower lobe, basal region
6.05 µm, VOI6	48.85	23.09	20.5	(−19.3, −4.9, 59.7)	0°	lower lobe, basal region
2.5 µm, VOI1	32	9.69	2.36	(18.4, −19.5, −84.5)	24.6°	upper lobe, apical region
2.5 µm, VOI2b	14	9.7	1.03	(0.3, −20.7, −83.6)	24.6°	upper lobe, apical region
2.5 µm, VOI3	14	9.69	1.03	(−12.6, −24.6, −65.7)	24.6°	upper lobe, apical region
2.5 µm, VOI4	14	9.69	1.03	(20.5, −19.0, −43.0)	24.6°	upper lobe, medial region
2.5 µm, VOI5	38.25	9.67	2.81	(−22.1, 6.4, 63.5)	24.6°	lower lobe, basal region
2.45 µm, VOI1	8.52	9.33	0.582	(−12.6, −23.7, −67.5)	4.8°	upper lobe, apical region
2.45 µm, VOI2	11.93	9.33	0.816	(−7.7, 12.0, 63.0)	2°	lower lobe, basal region
2.45 µm, VOI6	4.9	9.33	0.335	(−19.3, −4.9, 65.1)	0°	lower lobe, basal region

**Table 3:** Details of the hierarchical X-ray phase-contrast tomography data for a human left lung.

Folder name (.zip)	Binning	Image size	Voxel size (µm^3^)
25.08um_LADAF_2020-27_lung-left_pag-0.11_0.25_jp2_	1	5984 × 5984 × 8720	25.08^3^
50.16um_LADAF_2020-27_lung-left_pag-0.11_0.25_jp2_	2	2992 × 2992 × 4360	50.16^3^
100.32um_LADAF_2020-27_lung-left_pag-0.11_0.25_jp2_	4	1496 × 1496 × 2180	100.32^3^
6.5um_LADAF-2020-27_lung-left_VOI-01_pag-0.07_0.43_jp2_	1	3856 × 3856 × 7506	6.5^3^
13um_LADAF-2020-27_lung-left_VOI-01_pag-0.07_0.43_jp2_	2	1928 × 1928 × 3753	13.0^3^
6.5um_LADAF-2020-27_lung-left_VOI-02b_pag-0.12_0.45_jp2_	1	3854 × 3854 × 4114	6.5^3^
13um_LADAF-2020-27_lung-left_VOI-02b_pag-0.12_0.45_jp2_	2	1928 × 1928 × 3753	13.0^3^
6.5um_LADAF-2020-27_lung-left_VOI-03b_pag-0.12_0.46_jp2_	1	3852 × 3852 × 1402	6.5^3^
13um_LADAF-2020-27_lung-left_VOI-03b_pag-0.12_0.46_jp2_	2	1926 × 1926 × 701	13.0^3^
6.5um_LADAF-2020-27_lung-left_VOI-04b_pag-0.12_0.46_jp2_	1	3856 × 3856 × 16318	6.5^3^
13um_LADAF-2020-27_lung-left_VOI-04b_pag-0.12_0.46_jp2_	2	1928 × 1928 × 8159	13.0^3^
6.5um_LADAF-2020-27_lung-left_VOI-05_pag-0.08_0.43_jp2_	1	3852 × 3852 × 9202	6.5^3^
13um_LADAF-2020-27_lung-left_VOI-05_pag-0.08_0.43_jp2_	2	1926 × 1926 × 4601	13.0^3^
6.05um_LADAF-2020-27_lung-left_VOI-01_pag-0.01_0.03_jp2_	1	3822 × 3822 × 1258	6.05^3^
12.1um_LADAF-2020-27_lung-left_VOI-01_pag-0.01_0.03_jp2_	2	1911 × 1911 × 629	12.1^3^
6.05um_LADAF-2020-27_lung-left_VOI-02_pag-0.01_0.19_jp2_	1	3818 × 3818 × 6970	6.05^3^
12.1um_LADAF-2020-27_lung-left_VOI-02_pag-0.01_0.19_jp2_	2	1909 × 1909 × 3485	12.1^3^
6.05um_LADAF-2020-27_lung-left_VOI-06_pag-0.02_0.25_jp2_	1	3816 × 3816 × 8074	6.05^3^
12.1um_LADAF-2020-27_lung-left_VOI-06_pag-0.02_0.25_jp2_	2	1908 × 1908 × 4037	12.1^3^
2.5um_LADAF-2020-27_lung-left_ROI-01_pag-0.02_0.25_jp2_	1	3878 × 3878 × 12802	2.5^3^
5um_LADAF-2020-27_lung-left_ROI-01_pag-0.02_0.25_jp2_	2	1939 × 1939 × 6401	5.0^3^
2.5um_LADAF-2020-27_lung-left_VOI-02b_pag-0.04_0.34_jp2_	1	3880 × 3880 × 5600	2.5^3^
5um_LADAF-2020-27_lung-left_VOI-02b_pag-0.04_0.34_jp2_	2	1940 × 1940 × 2800	5.0^3^
2.5um_LADAF-2020-27_lung-left_VOI-03_pag-0.02_0.04_jp2_	1	3878 × 3878 × 5600	2.5^3^
5um_LADAF-2020-27_lung-left_VOI-03_pag-0.02_0.04_jp2_	2	1939 × 1939 × 2800	5.0^3^
2.5um_LADAF-2020-27_lung-left_VOI-04_pag-0.02_0.29_jp2_	1	3876 × 3876 × 5600	2.5^3^
5um_LADAF-2020-27_lung-left_VOI-04_pag-0.02_0.29_jp2_	2	1938 × 1938 × 2800	5.0^3^
2.5um_LADAF-2020-27_lung-left_VOI-05_pag-0.02_0.24_jp2_	1	3868 × 3868 × 15300	2.5^3^
5um_LADAF-2020-27_lung-left_VOI-05_pag-0.02_0.24_jp2_	2	1934 × 1934 × 7650	5.0^3^
2.45um_LADAF-2020-27_lung-left_01_pag-0.03_0.05_jp2_	1	3816 × 3816 × 3478	2.45^3^
4.9um_LADAF-2020-27_lung-left_01_pag-0.03_0.05_jp2_	2	1908 × 1908 × 1739	4.9^3^
2.45um_LADAF-2020-27_lung-left_02_pag-0.02_0.06_jp2_	1	3810 × 3810 × 4868	2.45^3^
4.9um_LADAF-2020-27_lung-left_02_pag-0.02_0.06_jp2_	2	1905 × 1905 × 999	4.9^3^
2.45um_LADAF-2020-27_lung-left_06_pag-0.02_0.04_jp2_	1	3810 × 3810 × 1998	2.45^3^
4.9um_LADAF-2020-27_lung-left_06_pag-0.02_0.04_jp2_	2	1905 × 1905 × 999	4.9^3^

### Volume selection and anatomical reference

**Fig. 3:**
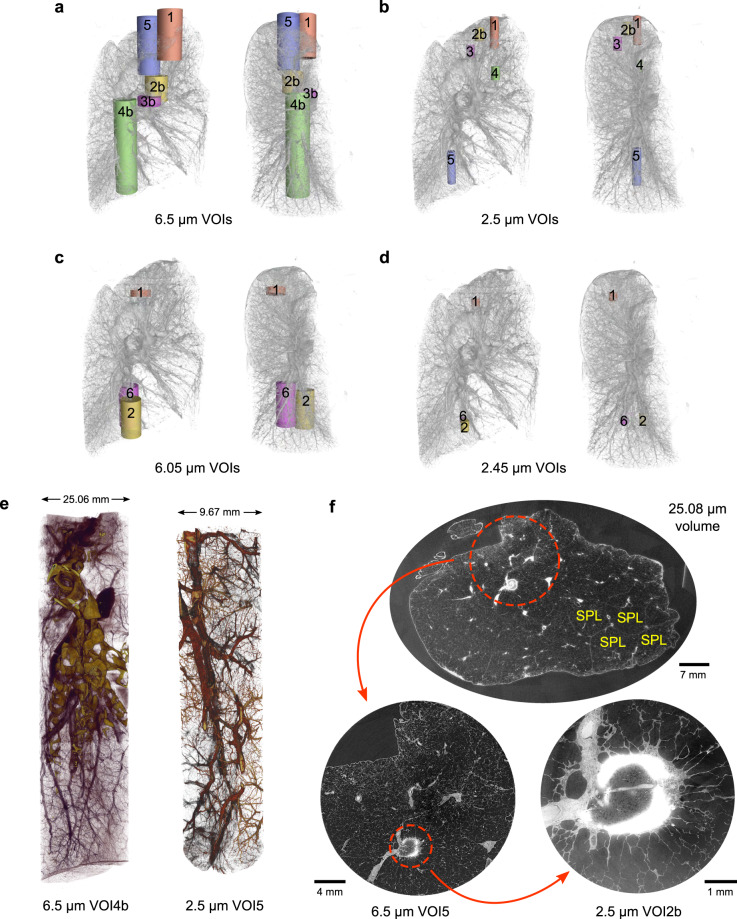
Exploration of the HiP-CT dataset of a human left lung. (**a**–**d**) Spatial correspondences of the measured cylindrical VOIs at different resolutions within the entire left lung. For each set of VOIs, both the medial (left) and sagittal (right) views are shown. The VOI label corresponds to the assignment in Table 2. (**e**) Renderings of two imaged VOIs with 6.5 *μ*m and 2.5 *μ*m voxel sizes, respectively. (**f**) From the whole lung and local tomography data, we visualize the anatomical detail of a partially calcified, spiculated lung nodule in the apical region of the lung on multiple lengthscales. The interlobular septa and perilobular vasculature of the secondary pulmonary lobules (SPLs) are depicted.

Besides the full-field tomography of the entire lung, subsequent smaller VOIs were selected with representative features and imaged with local tomography at higher resolution, including 6.5 *μ*m (5 locations) and 6.05 *μ*m (3 locations) for level 1 and 2.5 *μ*m (5 locations) and 2.45 *μ*m (3 locations) for level 2 VOIs, respectively. All VOIs have a cylindrical field of view around the rotation axis after removing the boundary artifacts from the local tomographic reconstruction. To obtain the displacements and rotations, the VOIs are spatially registered to the whole lung data by hand in VGStudio Max (version 3.4) and the procedure to apply them is described in Usage Notes. The sizes of the VOIs, their displacements, and rotations with respect to the center of the whole lung data are listed in Table 2 and illustrated in Fig. 3a–d. In addition, we provide brief anatomical references to the VOI spatial locations in Table 2 with respect to the whole lung data at 25.08 *μ*m. To retain traceable data provenance, we keep the same alphanumeric labels of the VOIs as used in the original experiments. Figure 3e visualizes two selected VOIs in the lower lobe of the lung.

## Data Records

We provide the volumetric data after reconstruction and post-processing as greyscale (16-bit) 2D image slices in the JPEG2000 format stored in zipped folders. The compression level of JPEG2000 is carefully chosen to ensure minimal difference from the original TIFF-formatted data when they are used for feature quantification or image segmentation. We list the details of the deposited data in Table 3. All data have been deposited at an ESRF data repository (https://human-organ-atlas.esrf.eu/explore/LADAF-2020-27/left%20lung) with digital object identifiers (DOIs) assigned to each scanned volume as listed in Table 4. Each DOI refers to a volume at full resolution and its binned versions. For all VOIs measured by local tomography, including those with voxel sizes of 6.5 *μ*m^44^–^48^, 6.05 *μ*m^49^–^51^, 2.5 *μ*m^52^–^56^, and 2.45 *μ*m^57^–^59^, both the full resolution data (Binning = 1) and the 2 × binned version (Binning = 2) are provided, while for the whole lung data^60^, the 4 × binned version (Binning = 4) is also provided. The metadata information in Table 2 is also provided in the corresponding text file contained in each data deposit. The landing page associated with each DOI contains extended information on the parameters of the X-ray beamline for phase-contrast tomography, experimental scanning protocol, and data processing procedures.

**Table 4:** Information about the data records.

Data description	Index	Reference
Full-field tomography data at 25.08 µm voxel size and binned versions (50.16 µm, 100.32 *µ*m voxel sizes)		^60^
Local tomography data at 6.5 µm voxel size and binned version (13.0 *µ*m voxel size)	VOI1	^44^
	VOI2b	^45^
	VOI3b	^46^
	VOI4b	^47^
	VOI5	^48^
Local tomography data at 6.05 µm voxel size and binned version (12.1 *µ*m voxel size)	VOI1	^49^
	VOI2	^50^
	VOI6	^51^
Local tomography data at 2.5 µm voxel size and binned version (5.0 *µ*m voxel size)	VOI1	^52^
	VOI2b	^53^
	VOI3	^54^
	VOI4	^55^
	VOI5	^56^
Local tomography data at 2.45 *µ*m voxel size and binned version (4.9 *µ*m voxel size)	VOI1	^57^
	VOI2	^58^
	VOI6	^59^

## Technical Validation

Although the radiation dose in HiP-CT scans is well below the tissue damage threshold^26^, due to radiation-induced bubble formation, which only appeared after multiple high-resolution, local tomography scans, the sample went through re-degassing before the remaining measurements were made. The bubbles largely come from solvent vaporization^61^ but don’t cause visible radiation damage as shown by histology^26^. However, a consequence of re-degassing is that not all of the VOIs have been imaged consecutively during the same beamtime. In the course of re-degassing, the sample was kept in the container to maintain its original position. The sample jar was then placed into the synchrotron X-ray beamline for further imaging. During the process, care was exercised such that the VOIs scanned before and after re-degassing can be registered to the whole volume without large deformation.

In the imaged volumes, contrast is produced by the local density differences between the lung tissue constituents and the void spaces of the airways, alveoli, and blood vessels filled with ethanol solution (see Fig. 3e,f). Within the whole lung data at a voxel size of 25.08 *μ*m, the interlobular septa, the boundaries of the secondary pulmonary lobules^62,63^ and the perilobular vasculature, are clearly visible (see Fig. 3f). At high spatial resolution, the local density difference increasingly becomes the dominant contributor to image contrast in VOIs^26^. The consistent contrast across lengthscales provides detailed information for the study of lung morphology for the healthy individual or as a control.

Regarding the total lung capacity for the individual, postmortem estimation is severely hampered by (1) the inability to directly measure anatomical dead space and (2) complex functional interactions between skeletal thorax, diaphragm configuration or tonus and pleural space, which are not reconstructible postmortem. Nevertheless, since the lung was inflated with near-normal pressure at fixation, our best assumption for near-normal inspiration inflation is pressure-controlled inflation. In addition, despite the great efforts in maintaining the integrity of the organ during preparation and scanning, in some subpleural areas of the lung, we have noticed a slight compression. We attribute the potential causes of these features to the following: (1) The formalin used in fixation may have difficulties in reaching the more peripheral lung areas. (2) The tight fit of the agar blocks around the lung. (3) A sign of minimal parabronchial and/or subpleural emphysema due to aging^5^.

## Usage Notes

The multiscale healthy human lung data presented here have been used as clinical control data in studies comparing damage within the lung microstructure due to Covid-19 infection^26^. The individual VOIs are deposited as 2D image slices perpendicular to the rotation axis (z in Fig. 1) in the tomography geometry. These images may be directly loaded into any typical image processing software for viewing or further quantification. To align the VOIs to the whole lung data, the following transform should be applied to the VOI,

1 $$I{\prime} (x,y,z)=T(dx,dy,dz){R}_{z}({\theta }_{z})I(x,y,z).$$

Here *I'* and *I* are intensity-valued volumetric data, *T* is the 3D translation operator, and *R*_*z*_ the 3D rotation operator around the *z* axis (see Fig. 1b,c). The displacement vector (*dx, dy, dz*) and *z* rotation angle *θ*_*z*_ for each VOI is listed in Table 2. The default greyscale ranges of the images are set with an intensity margin to avoid saturation. Viewing directly by eye may require threshold adjustment.

## Data Availability

The code used for the preprocessing, tomographic reconstruction, and postprocessing is available on GitHub (https://github.com/HiPCTProject/Tomo_Recon).
